# Diagnosis of Visceral Leishmaniasis in Bihar India: Comparison of the rK39 Rapid Diagnostic Test on Whole Blood Versus Serum

**DOI:** 10.1371/journal.pntd.0002233

**Published:** 2013-05-23

**Authors:** Greg Matlashewski, Vidya Nand Ravi Das, Krishna Pandey, Dharmendra Singh, Sushmita Das, Ayan Kumar Ghosh, Ravindra Nath Pandey, Pradeep Das

**Affiliations:** 1 Department of Microbiology and Immunology, McGill University, Montreal, Quebec, Canada; 2 Rajendra Memorial Research Institute of Medical Science, Patna, India; US Food and Drug Administration, United States of America

## Abstract

**Background:**

Antibody-detecting rapid diagnostic tests (RDTs) against rK39 are available to aid in the diagnosis of visceral leishmaniasis (VL). Although these rK39 RDTs have been developed, validated and approved for use with serum, they are universally performed using whole blood. It was therefore necessary to determine whether this RDT is as sensitive on whole blood as on serum.

**Method and Principal Findings:**

In this study we compared the rK39 RDT on serum and blood samples from 624 individuals with symptoms of VL attending the outpatient clinic at the Rajendra Memorial Research Institute of Medical Sciences, Patna, India. A total of 251 cases (40%) were both serum and blood-positive and 26 cases (4%) were identified as blood-negative and serum-positive. These 26 individuals in general had low titer antibodies against rK39 as determined by ELISA and follow-up on most of these individuals revealed none had persistent VL symptoms. The Cohen kappa index comparing blood and serum was 0.88 indicating excellent concordance.

**Conclusion:**

Although the concordance was excellent, it is possible to miss rK39 positive individuals when using blood and the titer of anti-rK39 antibodies is low. We recommend that when an individual from an endemic area has obvious clinical symptoms of VL and the whole blood rK39 RDT is negative, that the test should be redone 2–3 weeks later if the symptoms persist.

## Author Summary

Visceral leishmaniasis (VL), is a neglected tropical disease that is highly endemic in the Indian subcontinent and in East Africa and is the second most fatal parasitic disease after malaria. There currently exists several effective treatments for VL and it is therefore essential that the diagnosis be as accessible, sensitive and specific as possible. The current diagnostic test, known as the rK39 rapid diagnostic test (RDT) involves detection of antibodies against the K39 protein antigen from *Leishmania*. The rK39 RDT was developed for use with serum from potentially infected individuals. However, the test is routinely performed with blood at the community level in the endemic countries because there are no facilities to extract serum from blood. We therefore undertook the present study to compare the sensitivity of the rK39 RDT on serum versus blood from the same potentially infected population from a highly endemic region in Bihar India. Our results show that the concordance between serum and blood was excellent. It was however possible to miss some rK39 positive individuals when using blood. We recommend that when an individual from an endemic area has obvious clinical symptoms of VL and the blood rK39 RDT is negative, that the test should be redone 2–3 weeks later if the symptoms persist.

## Introduction

Visceral leishmaniasis (VL), also known as Kala-azar in the Indian subcontinent is a neglected tropical disease caused by *Leishmania donovani* (*L. donovani*) that is transmitted by the bite of an infected sandfly [1]. VL is highly endemic in the Indian subcontinent and in East Africa. Since VL is a fatal disease, it is essential that the diagnosis be as sensitive as possible. The main clinical features of VL are prolonged fever, anemia, splenomegaly, prominent wasting and when untreated death from organ failure and opportunistic infections [1]. There are an estimated 360000 new cases of VL each year world wide and over 60% of these occur in the Northern Indian state of Bihar and bordering regions of Nepal and Bangladesh [2]. There are now effective therapies to treat VL, including miltefosine, amphotericin B and liposomal amphotericin B that if used effectively could significantly reduce the burden of VL [3]. To reduce transmission, it is important to diagnose and treat cases as soon as possible since humans are the only reservoir for *L. donovani* in the Indian subcontinent [4].

The current method of VL diagnosis involved evaluating clinical symptom that include fever for more than 2 week, the presence of splenomegaly, and a positive serological rK39 immunochromatographic rapid diagnostic test (RDT) [1], [5]. The rK39 RDT is used to detect the presence of antibodies against the *Leishmania* antigen K39 that contains a repetitive 39 amino acid sequence from the kinesin protein. Clinical features of VL however can be mistaken for other febrile illnesses such as malaria and enteric fever. Therefore, accurate serological diagnosis with the rK39 RDT is essential. Although a number of rK39 RDTs are commercially available and have recently been shown to be effective on the Indian continent, these tests have been developed for use with serum [6]. These includes the Kalazar Detect test which, is the most widely used test in India. However, in order to be used at the point of care, the rK39 RDTs are routinely performed on blood instead of serum in the endemic regions of India, Nepal and Bangladesh [6]. It was therefore necessary in this study to establish whether the rK39 RDT is as sensitive when using blood as serum. This is a critical issue because performing the rK39 RDT on blood allows the test to be point of care at the level of primary health care centers close to the endemic villages, whereas performing the test on serum would require the test be performed at a district hospital which is generally much further from the endemic communities.

## Methods

### Patients

The study and informed consent forms were approved by the Rajendra Memorial Research Institute of Medical Sciences (RMRIMS) ethics review board. Parents provided written consent on behalf of participants under the age of 18. None of patients enrolled previously had VL or PKDL. Clinical suspicion for VL was defined as fever for more than 14 days and signs of splenomegaly. All suspected patients attended the out-patient department between August 2011 and April 2012.

### rK39 rapid test

The rK39 immunochromatographic RDT, Kalazar Detect (InBios International, USA) was performed at RMRIMS according to manufacturers instructions. At room temperature, 20 ul of serum prepared from venous blood or one drop of fingerstick blood was added to the dipstick. A single drop of blood was used in this study because this is what is routinely performed in the field. Three drops of the chase buffer solution was added to a test tube followed by addition of the dipstick into the test tube containing the chase buffer. The results were read after 10 minutes. The test was considered positive when both the control line and the test line appeared red in color. The level of agreement between the tests performed on serum versus blood was calculated using Cohen's kappa index.

### ELISA against recombinant K39 protein

Recombinant K39 protein was kindly provided by Dr. Steve Reed from the Infectious Diseases Research Institute, Seattle USA. Ninety six well microtiter plates were coated with 100 ul of 5 ug/ml rK39 in carbonate/bicarbonate buffer overnight. Wells were then washed extensively in 0.05% PBS-T and then blocked in 5% non fat dry milk +0.1% PBS-T for 1 h at 37°C followed by washing again in 0.05% PBS-T. Human sera samples (100 ul) at various dilution from 1∶50 to 1∶6400 was added for 2 hours at room temperature and then wells washed 3 times in 0.05% PBS-T. 100 uL of HRP-linked anti-human antibody (1∶5000) in 5% non fat dry milk +0.1% PBS/T was then added for 1 hr at room temperature followed by washing 4X with 0.05% PBS/Tween 20. Substrate (50 ul of TMB) was then added for 10 min at room temperature and the reaction stopped with 25 ul of 1 N H_2_SO_4_ and the optical density (OD) read at 450 nm. The significance of the data was evaluated by two-tailed Student's t test.

## Results

### Comparison of blood and serum using the rK39 RDT

A total of 624 patients attending the Rajendra Memorial Research Institute of Medical Sciences (RMRIMS) with symptoms of febrile splenomegaly were tested with the rK39 RDT (Kalazar Detect, InBios International) using serum and blood from the same patient. As shown in Table 1, 251 cases were seropositive on both blood and serum. In comparison, 334 were sero-negative on both serum and blood. Notably, there were 26 (4%) cases that were negative on blood and positive on serum. There were 13 cases that were positive on blood and negative on serum and these were considered to be false positives. This is consistent with a recent study using serum that reported the specificity of the rK39 RDT to be 96% when performed in India [6].

**Table 1:** Comparison of rK39 RDT on serum and blood from 624 cases with symptoms of febrile splenomegaly from Bihar.

Samples		Number	Percentage %
Blood (+)	Serum (+)	251	40
Blood (−)	Serum (−)	334	54
Blood (−)	Serum (+)	26	4
Blood (+)	Serum (−)	13	2
		Total: 624	100

Samples were obtained from patients attending the OPD clinic with symptoms of chronic fever and enlarged spleen were enrolled. Venous derived blood was used for serum preparation and whole blood was obtained by finger stick.

Compared to the results obtained with serum, the sensitivity (total positive/total positive + false negative) and specificity (total negative/total negative + false positive) of the test performed with blood was 91% and 93% respectively. Agreement of the rK39 RDT using serum versus blood resulted in a Cohen's kappa index of 0.88 (SE of kappa; 0.019), which demonstrates excellent concordance between these tests.

### ELISA against recombinant rK39 using serum and blood

**Figure 1:**
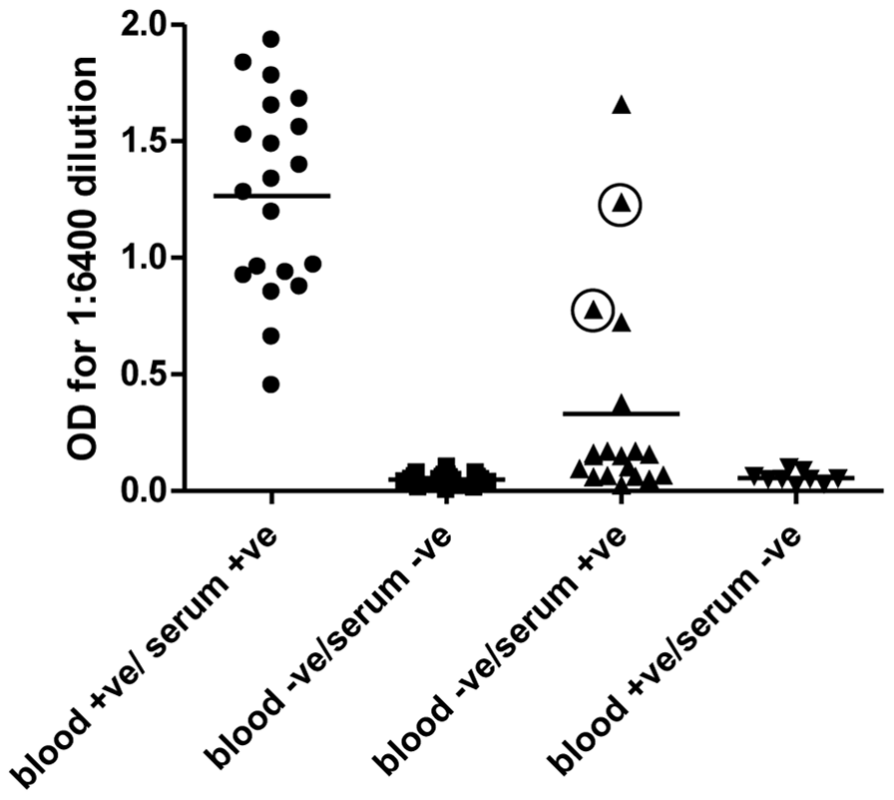
ELISA to determine relative anti-rK39 titers in serum sample. Serum samples were diluted at 1∶6400 dilution. The 2 circled samples in blood-negative, serum-positive samples were re-evaluated and found to be positive of the rK39 immunochromatographic RDT. Compared to Blood (−)/Serum (−), the difference with Blood (+)/Serum (+) was p = 2.3×10^−29^; the difference with Blood (−)/Serum (+) was p = 0.017; the difference Blood (+)/Seum (−) was p = 0.31.

The most important question from the rK39 RDT comparison of serum and blood was why 4% of the cases were negative on fingerstick blood, but were positive when tested with serum from the same patient. To investigate this question we performed an ELISA to determine the titer of the anti-rK39 antibodies from the different categories. The ELISA was performed with the rK39 recombinant protein on 20 samples from each category above accept for the Blood (+)/Serum (−) where there was only 13 samples. As shown in Figure 1, the titer of anti-rK39 antibodies in the blood-negative, serum-positive samples was much lower than for the Blood-positive, serum-positive samples but was significantly higher (p = 0.017) that the blood-negative, serum-negative samples. These observations demonstrate that samples can be interpreted as blood-negative and serum-positive when the titer of anti-rK39 antibodies is low. Finally, the titer of the blood-positive, serum-negative samples was similar to the blood-negative, serum-negative samples (p = 0.31) and these were considered to be false positives on blood.

## Discussion

Although the rK39 RDT has been evaluated and approved for use with serum, it is routinely used on whole blood at primary health centers and district hospitals in India, Nepal and Bangladesh. The major observation from this study is that the concordance of the rK39 RDT performed on serum versus blood is excellent (kappa = 0.88). The test can however be negative on blood and positive on serum when the titer of anti-K39 antibodies is low. We therefore recommend that when an individual from an endemic area has clinical symptoms of VL and the blood rK39 RDT is negative, that the test should be redone 2–3 weeks later if the symptoms persist since during this interval the titer may increase if the disease begins to progress.

In this study, none of the 26 cases that were blood-negative and serum-positive were treated for VL since the status of blood-negative is not indicated for treatment under the clinical practices at RMRIMS. This provided an opportunity to perform a follow-up on these patients of which 20 were found 3 to 6 months after attending the clinic either by direct observation or indirectly through discussion with relatives. None of the 20 cases identified had VL symptoms at follow-up nor had any of their family members developed symptoms of VL. This argues that, in this limited number, individuals with low titer anti-rK39 antibodies do not develop persistent VL symptoms. This is also consistent with previous observations that individuals with low titer antibodies to rK39 are less likely to have been treated or develop VL [7], [8]. Although this provides some reassurance that the majority of blood-negative and serum-positive did not require treatment, this does not rule out the possibility that VL can develop in some individuals with low-titer sera. These observations also support the argument that a diagnostic test that directly quantitates parasite levels in the blood could represent an important improvement for diagnosis of VL.

It was of interest to note that although the titer of the blood-negative, serum-positive samples was generally low there were several samples that had relatively high titers (Figure 1). The archived rK39 strips from these tests were therefore carefully re-examined. Remarkably, upon re-examination of the rK39 strips, 2 of these tests (Figure 1, shown with circles) did indeed have faint bands and were therefore positive yet were missed initially under routine screening conditions. In fact, the weak positive bands in these 2 test strips were the same intensity as the corresponding positive band on the test strip performed with serum. The difference between the blood and serum strips for these samples was that the strips using blood had a significant red background color (due to hemoglobin) that masked the weak band that is also red in color. None of the other blood-negative samples were positive under re-evaluation.

There is considerable variation in the background color for the samples when using blood. This is not the case when using serum which all displayed a white background making the serum samples easier to read. It is noteworthy that the instructions provided with the Kalazar Detect rK39 RDT do clearly state that serum should used instead of whole blood due to excessive background. This was the situation in the 2 cases from the blood-negative, serum-positive samples, which were initially misread in this study.

It is noteworthy that this study did not include microscopic detection of the amastigotes in spleen or bone marrow aspirates from patients as a reference standard. The major reasons were that the sensitivity of the microscopy when performed at RMRIMS is usually less than for the rK39 RDT and therefore is less than an ideal standard. It was also considered to be unethical to perform splenic or bone marrow aspirates on people who were rK39-negative. It was however possible to directly compare blood and serum in this study and this will help guide policy for VL elimination in the Indian sub-continent.
